# Bonding diversity in rock salt-type tellurides: examining the interdependence between chemical bonding and materials properties

**DOI:** 10.1039/d1ra02999a

**Published:** 2021-06-09

**Authors:** Jasmin Simons, Jan Hempelmann, Kai S. Fries, Peter C. Müller, Richard Dronskowski, Simon Steinberg

**Affiliations:** Institute of Inorganic Chemistry, RWTH Aachen University Landoltweg 1 D-52074 Aachen Germany simon.steinberg@ac.rwth-aachen.de; Jülich-Aachen Research Alliance (JARA-FIT and -HPC), RWTH Aachen University D-52056 Aachen Germany; Hoffmann Institute of Advanced Materials, Shenzhen Polytechnic 7098 Liuxian Blvd, Nanshan District Shenzhen China

## Abstract

Future technologies are in need of solid-state materials showing the desired chemical and physical properties, and designing such materials requires a proper understanding of their electronic structures. In this context, recent research on chalcogenides, which were classified as ‘incipient metals’ and included phase-change data storage materials as well as thermoelectrics, revealed a remarkable electronic behavior and possible state (dubbed ‘metavalency’) proposed for the frontier between entire electron localization and delocalization. Because the members of the family of the polar intermetallics vary widely in their properties as well as electronic structures, one may wonder if the aforementioned electronic characteristics are also achieved for certain polar intermetallics. To answer this question, we have employed quantum-chemical tools to examine the electronic structures of the rock salt-type YTe and SnTe belonging to the families of the polar intermetallics and incipient metals, respectively. To justify these classifications and argue as to why an application of the Zintl–Klemm concept (frequently employed to relate the structural features of tellurides to their electronic structures) could be misleading for YTe and SnTe, the electronic structures of YTe and SnTe were first compared to that of the rock salt-type SrTe. In addition, we carried out a *Gedankenexperiment* by subsequently modifying the chemical composition from YTe to SnTe, and, by doing so, we shed new light on the interdependence between chemical bonding and materials properties. Gradual changes in the former do not necessarily translate into the latter which may undergo discontinuous modifications.

## Abstract 2

Future technologies are in need of solid-state materials showing the desired chemical and physical properties, and designing such materials requires a proper understanding of their electronic structures.

## Graphical abstract

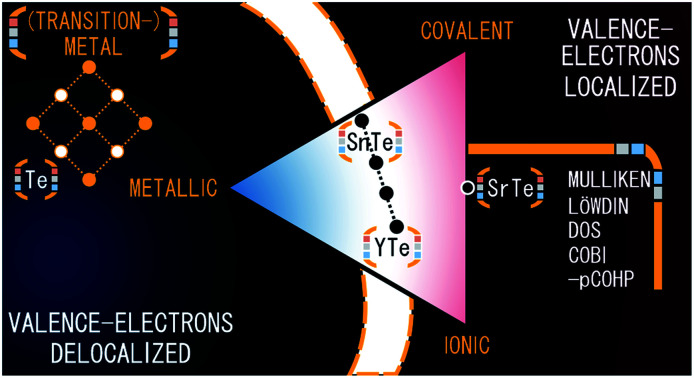

## Introduction

In light of the grand challenges in developing efficient and sustainable future technologies, there is a critical need^1^ to design (solid-state) materials serving as critical components. Among the broad and diverse realm of such materials, tellurides are of particular interest. Not only are several tellurides employed in existing technologies such as thermoelectrics^2–4^ or phase-change data storage devices,^5,6^ they are also at the forefront of basic research on, for instance, charge-density waves,^7,8^ superconductors^9,10^ or topological insulators.^11^ In general, the design of materials with desired chemical and physical properties also requires a proper understanding of their electronic structures.^12,13^ Some of the relationships between the crystal and the electronic structures in tellurides have typically^14,15^ been rationalized by applying the Zintl–Klemm idea, originally^16,17^ developed for dealing with intermetallics composed of main-group elements. In this framework,^18–20^ the valence-electrons are (formally) transferred from the less to the more electronegative elements, the latter arranged as clusters or fragments being isostructural to those observed for the isoelectronic elements. More recent efforts employing quantum-chemical techniques clearly corroborated such significant valence-electron transfers from alkali- and alkaline-earth metals to tellurides, thereby depicting the bonding nature as rather ionic^21,22^ (in full accordance with the Zintl–Klemm treatments). In the cases of the tellurides containing transition-metals, the bonding nature is better described as polar-covalent such that applying the Zintl–Klemm idea to such tellurides could be misleading.^23–28^

Within the most recent efforts on tellurides comprising post-transition-metals, a new bonding type dubbed ‘metavalent’^21,29^ or ‘hyperbonding’^30^ has been proposed. This type of bonding is expected to be at the frontier between entire valence-electron localization as well as delocalization and was introduced based on a portfolio of various quantities, seen both in experiment and calculation. The materials associated to this bonding type were classified as members of the family of the incipient metals^29^ containing a rich pool of phase-change data storage materials as well as thermoelectrics.^31–33^ It was also concluded that the degrees of sharing and transferring of valence-electrons should be decisive for reaching this particular electronic state; and yet, can such a remarkable electronic state also be accomplished for members of different families of solid-state materials? For instance, polar intermetallics, whose crystal structures are composed of polyanionic or polycationic fragments or clusters accompanied by monoatomic counterions, vary widely in their properties and show remarkable bonding situations, too, while not following any conventional valence-electron rules.^34^ To answer this question, we have carried out first-principles electronic-structure theory including a *Gedankenexperiment* in which the electronic structures of the polar intermetallic YTe, the incipient metal SnTe, and also the hypothetical “Y_1−*x*_Sn_*x*_Te” (*x* = 0.25, 0.75) were carefully varied and also studied by means of quantum-chemical techniques (please note that the quotation marks are used to denote hypothetical tellurides in the following). In doing so, we provide new insights into the interdependence between chemical bonding and materials properties for such materials. Prior to that, we also compared the electronic structures of SnTe and YTe to that of SrTe in order to justify the classifications of the former tellurides.

## Computational details

All quantum-chemical calculations included full optimizations of the lattice parameters and atomic positions for the inspected structure models by using the projector-augmented wave (PAW) method^35^ as implemented in the Vienna *ab initio* simulation package^36–40^ (VASP). Electronic correlation and exchange were described by the generalized gradient approximation of Perdew, Burke, and Enzerhof^41^ (GGA-PBE), while the plane-wave energy cut-off was set to 500 eV throughout for sets of 16 × 16 × 16 **k**-points in the first Brillouin zones, with convergence criteria below 10^−8^ (and 10^−6^) eV per cell for the electronic and (ionic) relaxation steps.

Bonding analyses were conducted based on projected crystal orbital Hamilton population (pCOHP) and their energy integrals (IpCOHP), the crystal orbital bond indices (COBI), and both Mulliken and Löwdin charges. The projected COHP^42^ is a modern variant of the traditional COHP technique in which the off-site densities-of-states are weighted by the respective Hamilton matrix elements to reveal bonding, nonbonding, and antibonding interactions.^43,44^ Because local basis sets are needed to detect the latter, all plane-wave-based (VASP) results must be unitarily transformed involving all-electron Slater-type orbitals. Likewise, the **k**-dependent density matrices served to compute the crystal orbital bond indices, solid-state analogues of the molecular bond order. The latter is obtained^45–48^ by summing over the square of all off-diagonal entries of its corresponding density matrix, while the solid-state COBIs are derived^49^ by summing over the square of all off-site entries of the respective **k**-dependent density matrix with respect to the band energy (see Appendix). In addition to the projected COHP and COBI, the atomic gross populations were also obtained from the plane waves to yield the Mulliken and Löwdin charges.^22^ All aforementioned calculations were employed by the Local Orbital Basis Suite Towards Electronic Structure Reconstruction code^42,43,50,51^ (LOBSTER). In general, it is non-trivial to directly compare (projected) COHPs and their integrals between different compounds, simply because the average electrostatic potentials in the DFT-based computations depend on arbitrary zero energies whose relative positions may vary from system to system. Hence, it is better to calculate the integrated values (total bonding) per cell and express all individual bonding interactions by their percentage contributions, as successfully done before.^44^

## Results and discussion

**Fig. 1:**
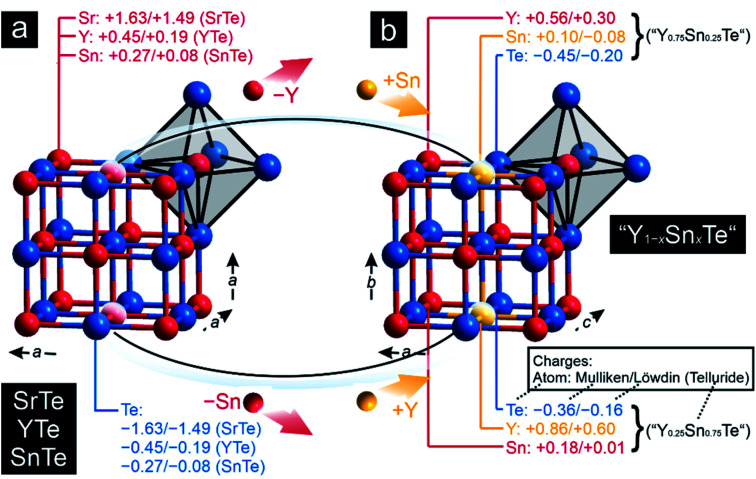
Representations of the crystal structures of (a) SrTe, SnTe, and YTe and (b) of “Y_0.75_Sn_0.25_Te” and “Y_0.25_Sn_0.75_Te”, whose crystal structures were derived from those of the former tellurides: the averaged Mulliken and Löwdin charges of the respective atoms have been included. All charges in units of *e*.

To explore the transition from the family of the polar intermetallics to that of the incipient metals, we first determined the electronic structures and bonding natures of YTe as well as SnTe, then compared them to SrTe because the valence-electron transfers may differ from a typical Zintl–Klemm case. Fig. 1 depicts their rock-salt crystal structure.

**Fig. 2:**
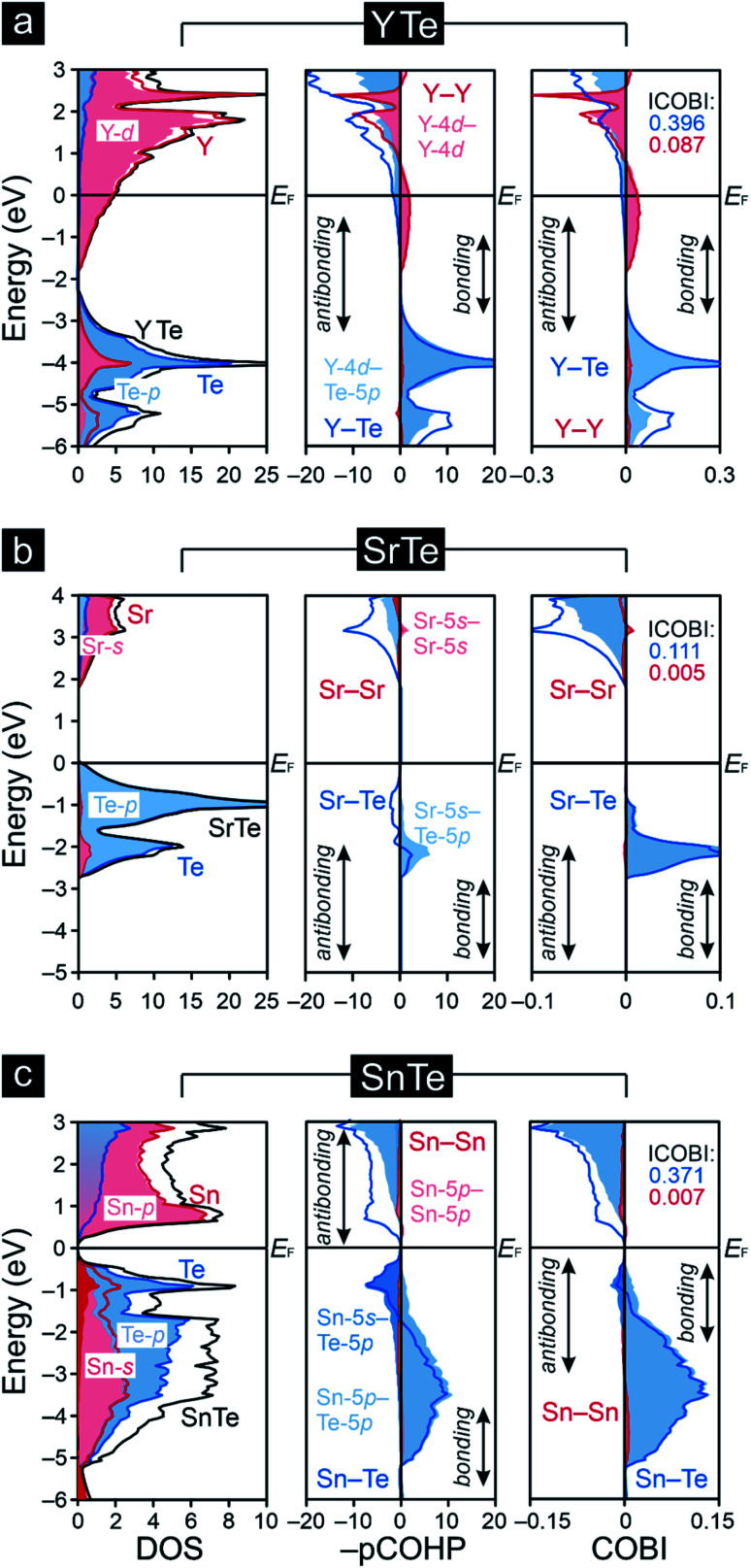
Densities-of-states (DOS), projected crystal orbital Hamilton populations (–pCOHP) and crystal orbital bond indices (COBI) of (a) YTe, (b) SrTe, and (c) SnTe: the Fermi levels, *E*_F_, are represented by the black horizontal lines, while the integrated COBI values (ICOBI) of the different interactions have been included.

The densities-of-states (DOS) curves of YTe, SrTe, and SnTe reveal that the Fermi levels fall in a wide band gap in SrTe and a narrow one in SnTe, while metallic YTe exhibits a significant DOS at *E*_F_ (Fig. 2). So, an electronically favorable situation^52^ is accomplished only for semiconducting SrTe and SnTe. Experimentally, YTe and SnTe are known as a metallic superconductor and a semiconductor, respectively.^33,53^ A closer look at the DOS of SrTe, YTe, and SnTe close to *E*_F_ demonstrates the Te-p character of the upper valence band, in addition to Y-d for YTe and Sn-s/p for SnTe. For SrTe, the Sr-s levels are way up in the conduction band, as expected^54,55^ for a fully oxidized alkaline-earth metal. For additional information, Fig. 1 provides Mulliken and Löwdin charges, and Fig. 2 also contains projected –COHP and COBI plots and also integrated values; integrated –pCOHP values are found in Table 1. Here and in the following, we focus on the levels close to the Fermi energy, as these states typically are most characteristic to the chemical bonds within a given compound. As there is a full valence-electron transfer from one atom to another one within an ionic bond, closed-shell species and a polar-attractive interaction will be evident.^56^ Such a bonding type is mirrored by valence-electron transfers close to the Zintl–Klemm values and smaller^57,58^ –IpCOHP and ICOBI values indicating less covalency. Somewhat simplified, covalent bonds are found whenever the valence-electrons are located between the interacting atoms for open-shell species insofar as more bonding states are filled than antibonding ones.^56^ Accordingly, covalent bonds correspond to no valence-electrons transfers (in contrast to the Zintl–Klemm ideal), but larger –IpCOHP and ICOBI values than for ionic bonds. In delocalized metal–metal bonds, there is also no or just a small valence-electron transfer (depending on the elements involved in a given metal–metal bonding); yet, the –IpCOHP and ICOBI values of the metal–metal bonds are smaller relative to those of covalent bonds due to the (delocalized) less bonding character of the former because of fewer electrons per bond.^57^ Under consideration of these general tendencies, we can now turn over to the results of the bonding analyses.

**Table 1:** Average –IpCOHP/bond values, cumulative –IpCOHP/cell values, and their percentage contributions to the net bonding capabilities for selected interactions in SrTe, YTe, and SnTe as well as the hypothetical “Sn_0.25_Y_0.75_Te” and “Y_0.25_Sn_0.75_Te”; the examinations of the diverse –pCOHP included those atomic orbitals providing the largest contributions to the states near the valence band maxima and minima

Interaction	Average –IpCOHP/bond (eV)	Cumulative –IpCOHP/cell (eV)	%
**SrTe**			
Sr-5s–Te-5p	0.1606	3.8550	89.69
Sr-5s–Sr-5s	0.0185	0.4432	10.31

**YTe**			
Y-4d–Te-5p	0.8723	20.9357	86.14
Y-4d–Y-4d	0.1404	3.3696	13.86

**SnTe**			
Sn-5s–Te-5p	0.1105	2.6508	9.30
Sn-5p–Te-5p	1.0476	25.1435	88.20
Sn-5p–Sn-5p	0.0297	0.7135	2.50

**“Sn**_**0.25**_ **Y**_**0.75**_ **Te”**			
Y-4d–Te-5p	0.8237	14.8269	60.50
Sn-5s–Te-5p	0.1482	0.8891	3.63
Sn-5p–Te-5p	1.0628	6.3766	26.02
Y-4d–Y-4d	0.1464	1.7563	7.16
Y-4d–Sn-5p	0.0549	0.6588	2.69

**“Y**_**0.25**_ **Sn**_**0.75**_ **Te”**			
Sn-5s–Te-5p	0.1180	2.1236	8.02
Sn-5p–Te-5p	1.0383	18.6897	70.56
Y-4d–Te-5p	0.7549	4.5292	17.10
Sn-5p–Sn-5p	0.0394	0.4725	1.78
Y-4d–Sn-5p	0.0562	0.6741	2.54

Clearly, the Mulliken and Löwdin charges for SrTe reveal that the valence-electron transfer from Sr to Te comes close to the Zintl–Klemm ideal of (Sr^2+^)(Te^2−^), the ionic case. Despite almost full Sr oxidation from the 5s levels, the cumulative –IpCOHP/cell value of the leftover Sr-5s–Sr-5s interactions given in Table 1 contribute 10.31% to the net bonding, because Sr-5s–Te-5p covalency is rather small, also mirrored from the Sr–Te ICOBI values in Fig. 2. The corresponding Mulliken and Löwdin charges of YTe, on the other side, clearly point to a small valence-electron transfer unlike that observed for the ionic SrTe, so the Y–Te contacts cannot be depicted as ionic. This outcome is in stark contrast to literature data^59–62^ suggesting ionic rare-earth–tellurium interactions, but agrees well with more recent research^23,25,26,28^ revealing polar-covalent bonding nature and suggesting the Zintl–Klemm formalism as potentially misleading. Examining the projected COHP of YTe brings to light that the largest bonding contribution originates from Y-4d–Te-5p which changes from bonding to antibonding below the Fermi level. These antibonding interactions are counterbalanced by homoatomic Y–Y interactions whose –IpCOHP/bond values are evidently smaller than those of the Y–Te contacts. Because there is no valence-electron transfer from one yttrium atom to another yttrium atom and the Y–Y separations (*d* = 4.362 Å) are longer than those distances typically^63^ observed for covalent Y–Y bonds, the here found homoatomic Y–Y interactions look like weak metal–metal bonds with an entirely delocalized character as well-known^58,64–66^ from reduced yttrium cluster halides and tellurides. In return, the Y–Te bonds correspond to a less delocalized character with a slight valence-electron transfer from the yttrium to the tellurium atoms (Fig. 1) and, accordingly, show a polar-covalent character as expected for rare-earth–tellurium contacts. This description is also mirrored by the COBIs and their integrated values (Fig. 2). The more covalent Y–Te interactions go by evidently larger ICOBI values than the more ionic Sr–Te interactions. As alluded to already, the Y–Y interactions are evidently weaker (ICOBI) than Y–Te contacts, and they are also mostly delocalized, as given by the itinerant DOS at *E*_F_. An alternative bonding picture of YTe would consist of an yttrium cluster (or bulk metal) partially oxidized by tellurium, as corroborated by previous research^67,68^ on transition-metal chalcogenides, another justification for assigning YTe to the family of the polar intermetallics. Because YTe comprises interactions in which the valence-electrons are both localized as well as delocalized (as shown by our bonding analyses), one may wonder if the remarkable electronic state at the frontier between electron localization and delocalization (see above) is also evident in YTe. Therefore, YTe was also considered to be predestinated for the search of materials which belong to different families and could exhibit the remarkable electronic state.

Previous research^69^ on rock salt-type chalcogenide-superconductors containing transition metals revealed that such metal–metal bonding and metal–chalcogenide antibonding near the Fermi levels play an important role in the occurrence of vacancies (which cannot be explained by applying any electron counting schemes). Namely, a subtle competition between two electronically unfavorable situations, *i.e.*, the presence of flat bands at *E*_F_ and populated antibonding levels, were found decisive. In this connection, it is remarkable that metal–metal bonding interactions, which could counterbalance the metal–chalcogenide antibonding interactions, are *not* evident for SnTe. While the transition metals' d orbitals can overlap and form metal–metal bonds, this is hardly the case for the Sn-5p orbitals. This is clearly seen from the Sn-5p–Sn-5p data in Table 1 and the tiny –IpCOHP values. Nonetheless, the occupied antibonding Sn–Te levels do correspond to an electronically unfavorable situation, and one may wonder if and how it may be alleviated. Indeed, previous research on post-transition-metal chalcogenides demonstrated that such change may occur by adopting a different type of structure,^70^ or by the depletions of such antibonding states through the introductions of vacancies.^71,72^ Notably, the presence of certain vacancies has also been identified^73^ for the crystal structure of SnTe. A closer inspection of the antibonding Sn–Te levels shows that they largely stem from interacting Sn-5s and Te-5p atomic orbitals. The small size of the Sn-5s–Te-5p values integrated over the entire energy range (Table 1) do not suggest that they can be neglected; instead, it is these atomic orbitals which make up antibonding close to *E*_F_. Although more recent research^74^ on post-transition-metal–chalcogenide bonding has mainly focused on the role of the interacting p-orbitals, it is obvious, both qualitatively and quantitatively, that Sn-5s–Te-5p bonding is an important aspect and must be included in order not to oversimplify the chemical bonding.

The small degree of charge transfer that is in good agreement with previous research^75,76^ on narrow-band-gap IV–VI semiconductors indicates a minor role of polar (or even ionic) bonding in SnTe. As said before, most bonding results from interacting Sn-5p and Te-5p atomic orbitals, despite characteristic antibonding below *E*_F_ where Sn-5s also mixes in. While there is some similarity even with YTe, the valence-electron transfer from Y to Te is larger than from Sn to Te, so the bonding nature of Y–Te differs from Sn–Te. Alternatively expressed, how can the Sn–Te ICOBI values, which are slightly smaller than those of Y–Te, barely attributable to polar contributions, be explained? Transitions from bonding to antibonding levels without any valence-electron transfers (like in metals)^77^ tend to position SnTe as approaching a metallic state, despite the narrow band gap, and the gap does not close because there is still a small charge transfer from Sn to Te, just like in other narrow-band-gap IV–VI semiconductors.^78^ In connection with previous Zintl–Klemm treatments of tellurides, the bonding of low-dimensional tellurium fragments undergoing structural distortions due to the formations of charge density waves was often^15,79^ interpreted in the light of solid-state hypervalency. Because the formations of charge density waves deal with the frontier between metallic and semiconducting states, one may wonder if this particular electronic state in SnTe could also be interpreted in terms of hypervalency; yet, the bonding situation in SnTe is far away from the Zintl–Klemm ideal, and an evidently polar bonding contribution being a characteristic of hypervalent bonding.^80,81^ From a more chemical perspective, it is remarkable that such solid-state materials simply do not follow the octet rule, a circumstance that is also evident for polar intermetallics like YTe.

**Fig. 3:**
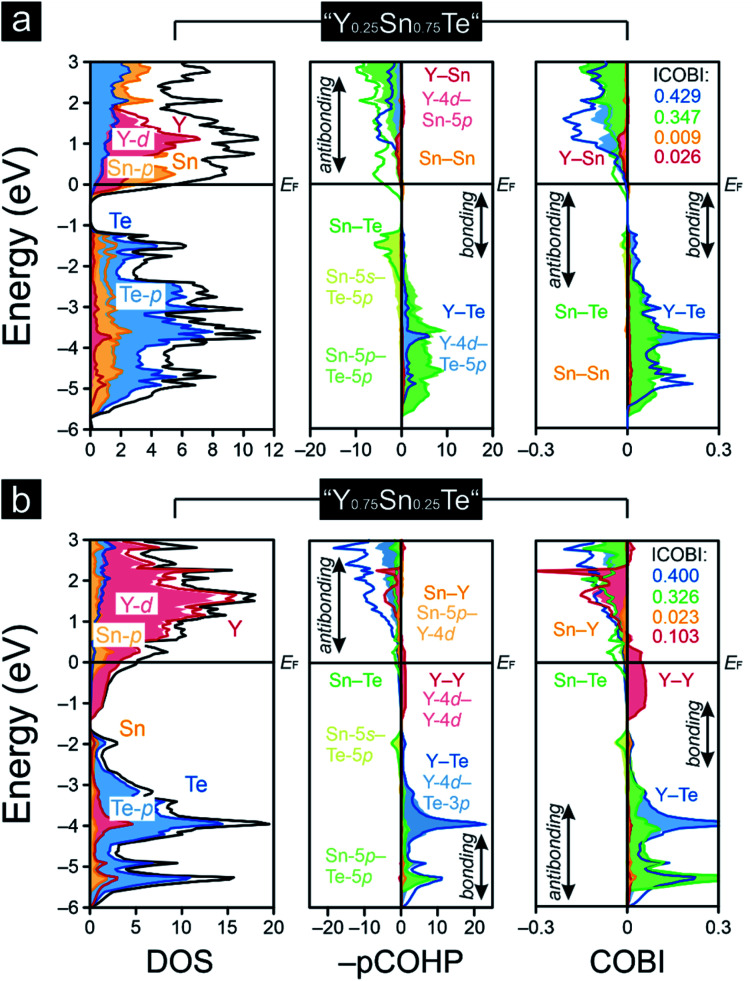
Densities-of-states (DOS), projected crystal orbital Hamiton populations (–pCOHP) and crystal orbital bond indices (COBI) of the hypothetical (a) “Y_0.25_Sn_0.75_Te” and (b) “Y_0.75_Sn_0.25_Te”; the Fermi level, *E*_F_, is represented by the black horizontal lines, while the integrated COBI (ICOBI) of the diverse interactions are included.

As pointed out before, YTe also undergoes a transition from a metallic to a superconducting states, while SnTe is located near the frontier between a metallic to semiconducting state. Hence, how will the transition between these remarkable electronic states look like for the series Y_1−*x*_Sn_*x*_Te? To answer this question, we carried out a *Gedankenexperiment* in which the gradual change of the electronic structures of SnTe and YTe were modelled by using two hypothetical tellurides, *i.e.* “Y_0.75_Sn_0.25_Te” and “Y_0.25_Sn_0.75_Te”. The rock-salt structure type (Fig. 1) was kept but exchanging one of either the Sn or Y atoms by each other, in harmony with experimental reports^82–84^ of solid solutions between SnTe and YTe. The calculated Mulliken and Löwdin charges of “Y_0.75_Sn_0.25_Te” and “Y_0.25_Sn_0.75_Te” yield that yttrium adopts a larger charge (gets even more cationic) in the ternary phase than in the binary. Likewise, tin is less strongly charged (less cationic) in the ternary than in the binary phase. That is to say that, upon moving from YTe to SnTe, yttrium is oxidized more while tin is reduced at the same time although there is no full charge transfer (needed for ionic bonding) from yttrium to tellurium. At the same time, there is almost no charge transfer from tin to tellurium, just like in the binary SnTe. The difference is given by the fact that the ternary tellurides become metallic (Fig. 3), unlike SnTe.

A comparison of the –IpCOHP/bond values (Table 1) of the hypothetical ternaries reveals that the Y–Te interactions correspond to smaller –IpCOHP/bond values than the Sn–Te interactions. The weaker bonding character of the former interactions appears to be an attribute of their more polar character relative to that of the Sn–Te bonds (as seen from the Mulliken and Löwdin population analyses). The charge analysis also indicates an absence of a full valence-electron transfer within the Y–Te bonds, suggesting that the Y–Te bonding in the ternary tellurides is similar to that in YTe, and it is also mirrored by the Y–Te ICOBI values: both in YTe and in the hypothetical tellurides they scale about in the same range, so the bonding nature must be quite similar, despite the fact that Y–Te could be slightly more polar in the ternaries than in YTe. For Sn–Te, things do not look that different. The Sn–Te ICOBI values in the ternaries are just slightly smaller than those in SnTe, so one may infer that Sn–Te bonding is also similar in all of the inspected tin-containing tellurides; yet, it is remarkable that the Sn–Te ICOBI values increase from “Y_0.75_Sn_0.25_Te” to SnTe, while the degree of valence-electron transfer from yttrium into the Sn–Te bonds decreases at the same time. Because the polarity of the Sn–Te bonds clearly decreases from SnTe to “Y_0.75_Sn_0.25_Te” (as indicated by the Mulliken and Löwdin charges), the decrease of the Sn–Te ICOBI values from SnTe to “Y_0.75_Sn_0.25_Te” must go back to increasing delocalization, certainly not to an enlarged polar character which does not exist. Nonetheless, let us reiterate that these bonding changes are rather subtle such that the overall bonding nature of the Sn–Te contacts is almost unaffected, despite the fact that the Fermi level characteristics of these tellurides are entirely different (Fig. 3). That is to say that the transition from a metallic to a semiconducting state appears as rather abrupt (like the metal-to-insulator-transitions induced by the formations of charge-density-waves^85^), while the changes in bonding nature look continuous and rather subtle within this series. Therefore, the series Y_1−*x*_Sn_*x*_Te, which has been studied to a lesser extent,^82,86–88^ appears to be an excellent candidate system to explore the influence of chemical compositions^89,90^ on the transition from a metallic to a semiconducting state. Clearly, the chemical composition influences the chemical bonding but compositional changes translate into rather small and, in particular, continuous changes in chemical bonding; for the physical properties, the changes may turn out as drastic, as a function of the respective electronic band structure.

## Conclusions

Understanding the electronic structures of solid-state materials is decisive because it provides invaluable information regarding the chemical and physical properties, in particular for the design of solid-state materials with tailored characteristics. In doing so, most recent research on tellurides proposed that some of them belong to the family of the incipient metals, which show an astonishing portfolio of properties associated with a particular electronic state. To probe if the properties of such materials can be fine-tuned by modifying the chemical composition, we used quantum-chemical means to analyze the electronic structures of the series Y_1−*x*_Sn_*x*_Te as it varies in a *Gedankenexperiment* between YTe and SnTe belonging to the families of the polar intermetallics and incipient metals, respectively. None of the two can be understood by applying simple valence-electron counting schemes.

To validate the aforementioned categories, the electronic structures of SrTe, YTe, and SnTe were analyzed in greater detail. The Sr–Te bonds are ionic, while entirely delocalized metal–metal bonding is evident for the Y–Y contacts in YTe. Neither Y–Te nor Sn–Te interactions are entirely ionic or delocalized but Y–Te can be safely described as being polar-covalent. The Sn–Te bonds are slightly less covalent than Y–Te, although the valence-electron transfer in the former is much smaller than in the latter. This lack in covalency for the Sn–Te bonds is attributable to their more delocalized nature, as also encountered for the multicenter bonds in metalloid^91,92^ clusters. As a narrow band gap opens at the Fermi level of SnTe, a metallic state is not accomplished. We conclude that the nature of Y–Te and Sn–Te bonding is very similar for the yttrium- and tin-containing tellurides, respectively, even including hypothetical ternaries, but it is the overall electronic band structures and their fillings which determine the respective Fermi levels, thereby determining the physical properties but not necessarily chemical properties for the entire Y_1−*x*_Sn_*x*_Te series. Hence, the transport properties cannot be traced back to the nature of the individual chemical bonds.

## Appendix

In the context of the bonding analyses of the herein reported tellurides, we also determined the crystal orbital bond indices (COBI). The COBI definition for periodic systems is derived from the molecular bond index (BI) by Wiberg and Mayer, which was originally based on the density matrices constructed for two-centered bonds between two atoms A and B
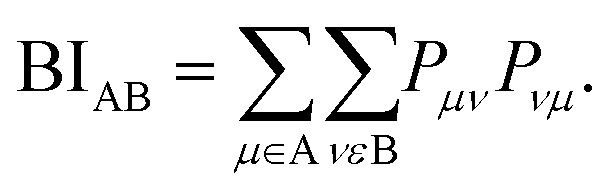


While this definition applies to basis sets employing atomic orbitals of the atoms A and B (within a molecule), the wave functions used to solve the Kohn–Sham equations for solid-state materials are typically constructed following Bloch's theorem. Taking this theorem into account, it is necessary to consider the **k**-dependence of the mixing coefficients *c*_*νi*_ and *c*_*μi*_ in order to get hold of the numbers of electrons occupying the crystal orbitals in a solid-state material. In doing so, the **k**-dependent density matrix that is weighted by the weighting factor *w*_*k*_ of each **k**-point is obtained. Furthermore, determining the electron numbers occupying the crystal orbitals also requires to consider the dependence of the (band) energy as included within the density-of-states-matrix by the band occupation *f*_*i*_
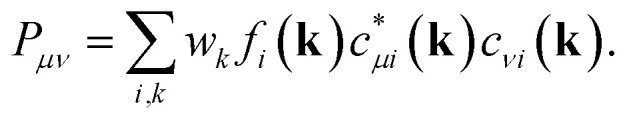


As shown^44^ for both COOP and COHP, the bonding information are solely obtained from the real parts of the (possibly) complex off-diagonal entries of the matrix such that just the real parts of the off-diagonal entries are included in the COBI, in which the density-of-states matrices replace the density matrices. The energy dependence is re-introduced by replacing the band occupation for one density-of-states matrix with a *δ*-distribution as it is done in the COOP and COHP approaches
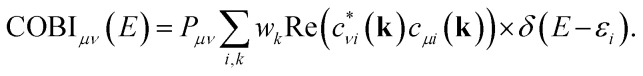


## Conflicts of interest

There are no conflicts of interest to declare.

## Supplementary Material
